# Poly[diaqua­bis­{μ-5-[4-(1*H*-imidazol-1-ylmethyl)phen­yl]tetra­zolato}copper(II)]

**DOI:** 10.1107/S1600536812015176

**Published:** 2012-04-18

**Authors:** Yuan Li, Ruizhan Chen

**Affiliations:** a Department of Chemistry, Changchun Normal University, Changchun 130032, People’s Republic of China

## Abstract

In the title compound, [Cu(C_11_H_9_N_6_)_2_(H_2_O)_2_]_*n*_, the Cu^II^ atom lies on an inversion center and is coordinated by four N atoms from four 5-[4-(1*H*-imidazol-1-ylmethyl)phen­yl]tetra­zolate ligands and two water mol­ecules in a distorted octa­hedral geometry. The ligands bridge the Cu^II^ atoms, leading to the formation of a two-dimensional network parallel to (100). The structure is further stabilized by O—H⋯N hydrogen bonds within the network.

## Related literature

For background to metal-organic architectures, see: Song *et al.* (2012 ▶); Wang *et al.* (2010 ▶). For background to metal-azolate frameworks, see: Masciocchi *et al.* (2005 ▶). For a related structure, see: Zhang *et al.* (2006 ▶).
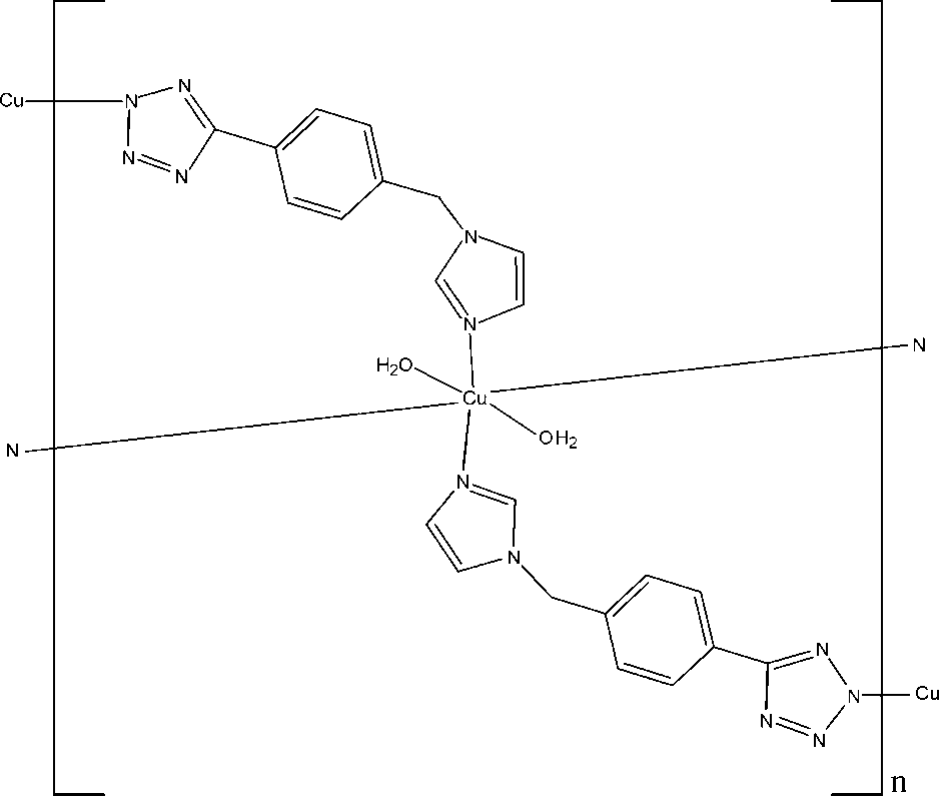


## Experimental

### 

#### Crystal data

[Cu(C_11_H_9_N_6_)_2_(H_2_O)_2_]
*M* *_r_* = 550.06
Monoclinic, 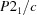

*a* = 7.3363 (10) Å
*b* = 6.1934 (9) Å
*c* = 25.219 (4) Å
β = 97.708 (2)°
*V* = 1135.5 (3) Å^3^
*Z* = 2
Mo *K*α radiation
μ = 1.01 mm^−1^
*T* = 293 K
0.25 × 0.21 × 0.20 mm

#### Data collection

Bruker APEXII CCD diffractometer
Absorption correction: multi-scan (*SADABS*; Bruker, 2001 ▶) *T*_min_ = 0.751, *T*_max_ = 0.824
6050 measured reflections
2224 independent reflections
2064 reflections with *I* > 2σ(*I*)
*R*_int_ = 0.015

#### Refinement

*R*[*F* ^2^ > 2σ(*F* ^2^)] = 0.031
*wR*(*F* ^2^) = 0.085
*S* = 1.09
2224 reflections
175 parameters
2 restraints
H atoms treated by a mixture of independent and constrained refinement
Δρ_max_ = 0.35 e Å^−3^
Δρ_min_ = −0.29 e Å^−3^

### 

Data collection: *APEX2* (Bruker, 2007 ▶); cell refinement: *SAINT* (Bruker, 2007 ▶); data reduction: *SAINT*; program(s) used to solve structure: *SHELXTL* (Sheldrick, 2008 ▶); program(s) used to refine structure: *SHELXTL*; molecular graphics: *XP* in *SHELXTL* and *DIAMOND* (Brandenburg, 1999 ▶); software used to prepare material for publication: *SHELXTL*.

## Supplementary Material

Crystal structure: contains datablock(s) global, I. DOI: 10.1107/S1600536812015176/hy2532sup1.cif

Structure factors: contains datablock(s) I. DOI: 10.1107/S1600536812015176/hy2532Isup2.hkl

Additional supplementary materials:  crystallographic information; 3D view; checkCIF report

## Figures and Tables

**Table 1:** Selected bond lengths (Å)

Cu1—N1	2.0247 (15)
Cu1—N6^i^	1.9909 (16)
Cu1—O1*W*	2.610 (2)

Symmetry code: (i) 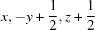.

**Table 2:** Hydrogen-bond geometry (Å, °)

*D*—H⋯*A*	*D*—H	H⋯*A*	*D*⋯*A*	*D*—H⋯*A*
O1*W*—H1*A*⋯N3^ii^	0.90 (2)	2.07 (2)	2.929 (3)	161 (3)

Symmetry code: (ii) 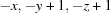.

**Fig. 1.**
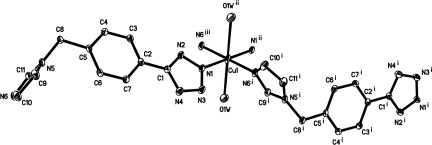
The asymmetric unit of the title compound. Displacement ellipsoids are drawn at the 30% probability level. H atoms have been omitted for clarity. [Symmetry codes: (i) x, 1/2-y, 1/2+z; (ii) -x, -y, 1-z; (iii) -x, -1/2+y, 1/2-z.]

**Fig. 2.**
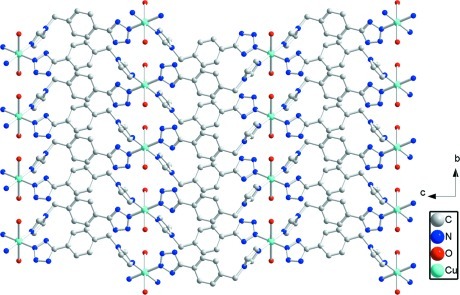
View of the two-dimensional network.

## supplementary crystallographic information

### Comment

Metal-organic architectures constructed by flexible, multifunctional ligands often exhibit structural diversity (Song *et al.*, 2012; Wang *et al.*, 2010). Metal-azolate frameworks, being composed of transition metal ions and deprotonated five-membered heterocycles, are regarded as a great achievement in understanding supramolecular isomerism (Masciocchi *et al.*, 2005). The azolate nitrogen donors can be presicely controlled as coordination and guest binding sites. In addition to the strong coordination ability toward transition metal ions, azolate ligands also combine the negative charge of carboxylates and predictable coordination modes of pyridines. Recently, we obtained the title complex by the reaction of copper chloride with 5-(4-imidazol-1-yl-benzyl)-2*H*-tetrazole using hydrothermal method and its crystal structure is reported here.

In the title compound, the Cu^II^ atom lies on an inversion center and adopts a distorted octahedral coordination geometry, being coordinated by four N atoms from four azolate ligands and two water molecules (Fig. 1, Table 1). The Cu—O and Cu—N bond lengths and the bond angles are in the normal range (Zhang *et al.*, 2006). The bridging azolate ligands allow the formation of a two-dimensional network parallel to (1 0 0) (Fig. 2). The crystal structure is further stabilized by O—H···N hydrogen bonds within the network (Table 2).

### Experimental

A mixture of CuCl_2_.2H_2_O (0.2 mmol, 0.034 g), 5-(4-imidazol-1-yl-benzyl)-2*H*-tetrazole (0.2 mmol, 0.045 g), NaOH (0.2 mmol, 0.008 g) and water (10 ml) was sealed in a 15 ml Teflon-lined reactor, which was heated at 120°C for 72 h and then gradually cooled to room temperature. Blue crystals were obtained.

### Refinement

H atoms on C atoms were generated geometrically and refined as riding atoms, with C—H = 0.93 (aromatic) and 0.97 (CH_2_) Å and *U*_iso_(H) = 1.2*U*_eq_(C). Water H atoms were located in a difference Fourier map and refined with *U*_iso_(H) = 1.5*U*_eq_(O).

### Crystal data

**Table:**

[Cu(C_11_H_9_N_6_)_2_(H_2_O)_2_]	*F*(000) = 566
*M**_r_* = 550.06	*D*_x_ = 1.609 Mg m^−^^3^
Monoclinic, *P*2_1_/*c*	Mo *K*α radiation, λ = 0.71073 Å
Hall symbol: -P 2ybc	Cell parameters from 2224 reflections
*a* = 7.3363 (10) Å	θ = 1.0–26.0°
*b* = 6.1934 (9) Å	µ = 1.01 mm^−^^1^
*c* = 25.219 (4) Å	*T* = 293 K
β = 97.708 (2)°	Block, blue
*V* = 1135.5 (3) Å^3^	0.25 × 0.21 × 0.20 mm
*Z* = 2	

### Data collection

**Table:**

Bruker APEXII CCD diffractometer	2224 independent reflections
Radiation source: fine-focus sealed tube	2064 reflections with *I* > 2σ(*I*)
Graphite monochromator	*R*_int_ = 0.015
φ and ω scans	θ_max_ = 26.0°, θ_min_ = 1.6°
Absorption correction: multi-scan (*SADABS*; Bruker, 2001)	*h* = −6→9
*T*_min_ = 0.751, *T*_max_ = 0.824	*k* = −7→7
6050 measured reflections	*l* = −27→31

### Refinement

**Table:**

Refinement on *F*^2^	Primary atom site location: structure-invariant direct methods
Least-squares matrix: full	Secondary atom site location: difference Fourier map
*R*[*F*^2^ > 2σ(*F*^2^)] = 0.031	Hydrogen site location: inferred from neighbouring sites
*wR*(*F*^2^) = 0.085	H atoms treated by a mixture of independent and constrained refinement
*S* = 1.09	*w* = 1/[σ^2^(*F*_o_^2^) + (0.044*P*)^2^ + 0.7185*P*] where *P* = (*F*_o_^2^ + 2*F*_c_^2^)/3
2224 reflections	(Δ/σ)_max_ < 0.001
175 parameters	Δρ_max_ = 0.35 e Å^−^^3^
2 restraints	Δρ_min_ = −0.29 e Å^−^^3^

### Special details

**Table:**

Geometry. All e.s.d.'s (except the e.s.d. in the dihedral angle between two l.s. planes) are estimated using the full covariance matrix. The cell e.s.d.'s are taken into account individually in the estimation of e.s.d.'s in distances, angles and torsion angles; correlations between e.s.d.'s in cell parameters are only used when they are defined by crystal symmetry. An approximate (isotropic) treatment of cell e.s.d.'s is used for estimating e.s.d.'s involving l.s. planes.
Refinement. Refinement of *F*^2^ against ALL reflections. The weighted *R*-factor *wR* and goodness of fit *S* are based on *F*^2^, conventional *R*-factors *R* are based on *F*, with *F* set to zero for negative *F*^2^. The threshold expression of *F*^2^ > σ(*F*^2^) is used only for calculating *R*-factors(gt) *etc*. and is not relevant to the choice of reflections for refinement. *R*-factors based on *F*^2^ are statistically about twice as large as those based on *F*, and *R*- factors based on ALL data will be even larger.

### Fractional atomic coordinates and isotropic or equivalent isotropic displacement parameters (Å^2^)

**Table:**

	*x*	*y*	*z*	*U*_iso_*/*U*_eq_	
Cu1	0.0000	0.0000	0.5000	0.03061 (14)	
C1	0.1942 (2)	0.1287 (3)	0.35950 (7)	0.0210 (4)	
C2	0.2239 (2)	0.0738 (3)	0.30431 (7)	0.0206 (4)	
C3	0.1855 (3)	−0.1330 (3)	0.28390 (7)	0.0238 (4)	
H3	0.1428	−0.2387	0.3054	0.029*	
C4	0.2110 (3)	−0.1813 (3)	0.23170 (7)	0.0241 (4)	
H4	0.1839	−0.3193	0.2183	0.029*	
C5	0.2764 (2)	−0.0262 (3)	0.19905 (7)	0.0204 (4)	
C6	0.3139 (3)	0.1795 (3)	0.21932 (7)	0.0246 (4)	
H6	0.3571	0.2848	0.1978	0.030*	
C7	0.2875 (3)	0.2295 (3)	0.27156 (7)	0.0235 (4)	
H7	0.3126	0.3682	0.2847	0.028*	
C8	0.3083 (3)	−0.0898 (3)	0.14277 (7)	0.0252 (4)	
H8A	0.4269	−0.1604	0.1444	0.030*	
H8B	0.2147	−0.1928	0.1285	0.030*	
C9	0.1502 (3)	0.1771 (3)	0.07892 (7)	0.0252 (4)	
H9	0.0324	0.1233	0.0800	0.030*	
C10	0.3754 (3)	0.3717 (4)	0.06022 (8)	0.0337 (5)	
H10	0.4416	0.4790	0.0454	0.040*	
C11	0.4493 (3)	0.2182 (4)	0.09521 (8)	0.0315 (5)	
H11	0.5727	0.2005	0.1087	0.038*	
N1	0.1185 (2)	0.1067 (3)	0.43685 (6)	0.0249 (3)	
N2	0.1231 (3)	−0.0072 (2)	0.39174 (7)	0.0287 (4)	
N3	0.1844 (2)	0.3016 (3)	0.43178 (6)	0.0293 (4)	
N4	0.2332 (3)	0.3209 (3)	0.38258 (7)	0.0316 (4)	
N5	0.3034 (2)	0.0945 (3)	0.10656 (6)	0.0230 (3)	
N6	0.1884 (2)	0.3443 (3)	0.05002 (6)	0.0273 (4)	
O1W	−0.2368 (3)	0.3145 (3)	0.50004 (7)	0.0427 (4)	
H1A	−0.214 (4)	0.447 (3)	0.5139 (12)	0.064*	
H1B	−0.336 (3)	0.265 (5)	0.5113 (12)	0.064*	

### Atomic displacement parameters (Å^2^)

**Table:**

	*U*^11^	*U*^22^	*U*^33^	*U*^12^	*U*^13^	*U*^23^
Cu1	0.0456 (2)	0.0346 (2)	0.01278 (19)	−0.02094 (15)	0.00818 (14)	−0.00562 (13)
C1	0.0209 (9)	0.0248 (9)	0.0174 (9)	−0.0010 (7)	0.0029 (7)	−0.0002 (7)
C2	0.0200 (8)	0.0262 (9)	0.0160 (9)	0.0007 (7)	0.0041 (6)	0.0006 (7)
C3	0.0276 (9)	0.0250 (10)	0.0195 (9)	−0.0025 (8)	0.0062 (7)	0.0033 (7)
C4	0.0296 (10)	0.0230 (9)	0.0198 (9)	−0.0006 (8)	0.0036 (7)	−0.0006 (7)
C5	0.0206 (9)	0.0264 (9)	0.0139 (9)	0.0048 (7)	0.0012 (7)	0.0025 (7)
C6	0.0308 (10)	0.0270 (10)	0.0170 (9)	−0.0009 (8)	0.0067 (7)	0.0058 (7)
C7	0.0289 (10)	0.0227 (9)	0.0188 (9)	−0.0029 (8)	0.0034 (7)	−0.0007 (7)
C8	0.0345 (10)	0.0261 (10)	0.0153 (9)	0.0079 (8)	0.0043 (7)	0.0033 (7)
C9	0.0283 (10)	0.0286 (10)	0.0185 (9)	0.0068 (8)	0.0030 (7)	0.0025 (7)
C10	0.0367 (11)	0.0403 (12)	0.0265 (11)	0.0037 (9)	0.0136 (9)	0.0104 (9)
C11	0.0276 (10)	0.0408 (12)	0.0271 (10)	0.0025 (9)	0.0070 (8)	0.0062 (9)
N1	0.0346 (9)	0.0250 (8)	0.0160 (7)	−0.0079 (7)	0.0064 (6)	−0.0040 (6)
N2	0.0454 (11)	0.0258 (9)	0.0167 (8)	−0.0091 (7)	0.0110 (7)	−0.0039 (6)
N3	0.0429 (10)	0.0269 (9)	0.0200 (8)	−0.0103 (7)	0.0116 (7)	−0.0042 (6)
N4	0.0479 (11)	0.0281 (9)	0.0217 (8)	−0.0110 (8)	0.0149 (7)	−0.0035 (7)
N5	0.0276 (8)	0.0282 (8)	0.0134 (7)	0.0056 (7)	0.0039 (6)	0.0024 (6)
N6	0.0362 (9)	0.0324 (9)	0.0142 (7)	0.0107 (7)	0.0067 (6)	0.0041 (6)
O1W	0.0577 (11)	0.0355 (9)	0.0361 (9)	−0.0071 (8)	0.0100 (8)	−0.0050 (7)

### Geometric parameters (Å, º)

**Table:**

Cu1—N1	2.0247 (15)	C8—N5	1.459 (2)
Cu1—N6^i^	1.9909 (16)	C8—H8A	0.9700
Cu1—O1W	2.610 (2)	C8—H8B	0.9700
C1—N2	1.325 (2)	C9—N6	1.318 (3)
C1—N4	1.339 (2)	C9—N5	1.341 (2)
C1—C2	1.477 (2)	C9—H9	0.9300
C2—C7	1.391 (3)	C10—C11	1.359 (3)
C2—C3	1.395 (3)	C10—N6	1.372 (3)
C3—C4	1.387 (3)	C10—H10	0.9300
C3—H3	0.9300	C11—N5	1.378 (3)
C4—C5	1.392 (3)	C11—H11	0.9300
C4—H4	0.9300	N1—N3	1.313 (2)
C5—C6	1.386 (3)	N1—N2	1.343 (2)
C5—C8	1.521 (2)	N3—N4	1.342 (2)
C6—C7	1.392 (3)	O1W—H1A	0.900 (18)
C6—H6	0.9300	O1W—H1B	0.874 (18)
C7—H7	0.9300		
			
N6^ii^—Cu1—N6^i^	180.00	C2—C7—H7	119.7
N6^ii^—Cu1—N1	89.67 (6)	C6—C7—H7	119.7
N6^i^—Cu1—N1	90.33 (6)	N5—C8—C5	112.80 (15)
N6^ii^—Cu1—N1^iii^	90.33 (6)	N5—C8—H8A	109.0
N6^i^—Cu1—N1^iii^	89.67 (6)	C5—C8—H8A	109.0
N1—Cu1—N1^iii^	180.0	N5—C8—H8B	109.0
O1W—Cu1—N1	96.47 (6)	C5—C8—H8B	109.0
O1W—Cu1—N6^ii^	87.49 (7)	H8A—C8—H8B	107.8
O1W—Cu1—O1W^iii^	180.00	N6—C9—N5	111.21 (18)
O1W—Cu1—N1^iii^	83.53 (6)	N6—C9—H9	124.4
O1W—Cu1—N6^i^	92.51 (7)	N5—C9—H9	124.4
N2—C1—N4	112.13 (16)	C11—C10—N6	109.69 (18)
N2—C1—C2	123.50 (16)	C11—C10—H10	125.2
N4—C1—C2	124.37 (16)	N6—C10—H10	125.2
C7—C2—C3	119.01 (16)	C10—C11—N5	105.68 (18)
C7—C2—C1	120.23 (17)	C10—C11—H11	127.2
C3—C2—C1	120.75 (16)	N5—C11—H11	127.2
C4—C3—C2	120.16 (17)	N3—N1—N2	110.41 (15)
C4—C3—H3	119.9	N3—N1—Cu1	125.39 (12)
C2—C3—H3	119.9	N2—N1—Cu1	123.89 (12)
C3—C4—C5	120.89 (17)	C1—N2—N1	104.08 (15)
C3—C4—H4	119.6	N1—N3—N4	108.63 (15)
C5—C4—H4	119.6	C1—N4—N3	104.76 (15)
C6—C5—C4	118.91 (16)	C9—N5—C11	107.46 (16)
C6—C5—C8	122.29 (16)	C9—N5—C8	124.89 (16)
C4—C5—C8	118.79 (16)	C11—N5—C8	127.63 (16)
C5—C6—C7	120.50 (17)	C9—N6—C10	105.94 (16)
C5—C6—H6	119.8	C9—N6—Cu1^iv^	123.39 (14)
C7—C6—H6	119.8	C10—N6—Cu1^iv^	130.58 (14)
C2—C7—C6	120.53 (17)	H1A—O1W—H1B	109 (3)
			
N2—C1—C2—C7	175.61 (18)	N6^i^—Cu1—N1—N2	146.67 (17)
N4—C1—C2—C7	−3.5 (3)	N4—C1—N2—N1	−0.1 (2)
N2—C1—C2—C3	−3.2 (3)	C2—C1—N2—N1	−179.28 (17)
N4—C1—C2—C3	177.71 (18)	N3—N1—N2—C1	−0.2 (2)
C7—C2—C3—C4	0.0 (3)	Cu1—N1—N2—C1	173.62 (13)
C1—C2—C3—C4	178.88 (17)	N2—N1—N3—N4	0.4 (2)
C2—C3—C4—C5	0.6 (3)	Cu1—N1—N3—N4	−173.29 (13)
C3—C4—C5—C6	−0.8 (3)	N2—C1—N4—N3	0.3 (2)
C3—C4—C5—C8	177.86 (17)	C2—C1—N4—N3	179.51 (17)
C4—C5—C6—C7	0.3 (3)	N1—N3—N4—C1	−0.4 (2)
C8—C5—C6—C7	−178.27 (17)	N6—C9—N5—C11	0.8 (2)
C3—C2—C7—C6	−0.5 (3)	N6—C9—N5—C8	179.32 (16)
C1—C2—C7—C6	−179.34 (17)	C10—C11—N5—C9	−0.5 (2)
C5—C6—C7—C2	0.3 (3)	C10—C11—N5—C8	−178.94 (17)
C6—C5—C8—N5	−24.7 (3)	C5—C8—N5—C9	−85.6 (2)
C4—C5—C8—N5	156.69 (17)	C5—C8—N5—C11	92.7 (2)
N6—C10—C11—N5	0.0 (2)	N5—C9—N6—C10	−0.8 (2)
N6^ii^—Cu1—N1—N3	139.55 (17)	N5—C9—N6—Cu1^iv^	176.09 (12)
N6^i^—Cu1—N1—N3	−40.45 (17)	C11—C10—N6—C9	0.5 (2)
N6^ii^—Cu1—N1—N2	−33.33 (17)	C11—C10—N6—Cu1^iv^	−176.08 (14)

Symmetry codes: (i) *x*, −*y*+1/2, *z*+1/2; (ii) −*x*, *y*−1/2, −*z*+1/2; (iii) −*x*, −*y*, −*z*+1; (iv) −*x*, *y*+1/2, −*z*+1/2.

### Hydrogen-bond geometry (Å, º)

**Table:**

*D*—H···*A*	*D*—H	H···*A*	*D*···*A*	*D*—H···*A*
O1*W*—H1*A*···N3^v^	0.90 (2)	2.07 (2)	2.929 (3)	161 (3)

Symmetry code: (v) −*x*, −*y*+1, −*z*+1.
